# Extended-spectrum β-lactamase-producing and carbapenemase-producing *Enterobacteriaceae*

**DOI:** 10.1099/mgen.0.000197

**Published:** 2018-07-23

**Authors:** Hayley Wilson, M. Estée Török

**Affiliations:** ^1^​ Department of Medicine, University of Cambridge, Addenbrooke's Hospital, Cambridge CB2 0QQ, UK; ^2^​ Cambridge University Hospitals NHS Foundation Trust, Cambridge, UK; ^3^​ Clinical Microbiology and Public Health Laboratory, Public Health England, Cambridge, UK

**Keywords:** ESBL, CPE, antimicrobial resistance, Gram-negative, *Enterobacteriaceae*

## Abstract

Antimicrobial resistance (AMR) is a global public-health emergency, which threatens the advances made by modern medical care over the past century. The World Health Organization has recently published a global priority list of antibiotic-resistant bacteria, which includes extended-spectrum β-lactamase-producing *Enterobacteriaceae* and carbapenemase-producing *Enterobacteriaceae*. In this review, we highlight the mechanisms of resistance and the genomic epidemiology of these organisms, and the impact of AMR.

**Impact Statement**

The World Health Organization (WHO) has published a global priority pathogens list of antibiotic-resistant bacteria, in order to increase the significance of and galvanize research into new treatments for particular antibiotic-resistant pathogens. Of critical importance on this list are carbapenem-resistant *Acinetobacter baumanii, Pseudomonas aeruginosa* and *Enterobacteriaceae*. Pathogens of this nature cause high morbidity and mortality and increased healthcare costs. Carbapenem-resistant *Enterobacteriaceae* encompasses a number of genera, many of which harbour acquired multidrug-resistance plasmids, which can carry and transmit antimicrobial-resistance genes on an intra- and inter-species level. This complicates surveillance, outbreak investigations and actions by infection control professionals. The spread of multidrug resistance is a globally important problem, with several countries currently reporting endemicity of highly resistant pathogens such as carbapenem-resistant *Klebsiella pneumoniae*. We have reviewed the current literature on carbapenem and third-generation cephalosporin-resistant *Enterobacteriaceae*. Our review highlights the continued increasing trend of resistance in *Enterobacteriaceae* and discusses the mechanisms by which this occurs. We aim to provide valuable collated information as part of a series of reviews on the WHO priority pathogens and enhance the current understanding in this area.

## Introduction

The development and introduction of antimicrobials in the 20th century has transformed the delivery of modern medical care. Yet, this ‘antibiotic golden-age’ is ending, threatened by rising rates of antimicrobial resistance (AMR) globally. *Enterobacteriaceae*, a family encompassing many clinically important bacterial species, exhibits rising levels of AMR. Infection with either extended-spectrum β-lactamase-producing *Enterobacteriaceae* (ESBL-E) or carbapenemase-producing *Enterobacteriaceae* (CPE) is associated with increased mortality rates, time to effective therapy, length of stay and overall healthcare costs [1–8]. The impact of the continued spread of AMR could have repercussions in multiple sectors. In the healthcare sector itself, patient deaths resulting from AMR are projected to reach 10 million annually by 2050, but AMR will also cause losses in the trillions to global economic output [9]. ESBL-E and CPE have spread globally [10, 11], and technologies such as whole-genome sequencing (WGS) are providing detailed insights into their evolution and dissemination. The World Health Organization has recently published a global priority pathogens list to focus attention on the most significantly resistant pathogens. *Enterobacteriaceae* resistant to third-generation cephalosporins (which includes ESBL-E) and *Enterobacteriaceae* resistant to carbapenems (CRE) are included within the critical category of this list [12].

## ESBL-E

The definition of multidrug resistance is variable [13], but *Enterobacteriaceae* exhibiting resistance to β-lactams, extended-spectrum β-lactams and third-generation cephalosporins are commonly recognized as ESBL-E [11, 14]. Extended-spectrum β-lactamase (ESBL) mechanisms themselves are classified based on their molecular structure or functional similarities [15, 16] (Table 1). Initially, ESBL-E were predominantly associated with nosocomial outbreaks, with resistance arising from point mutations in plasmid-mediated enzymes such as TEM-1, TEM-2, SHV-1 and OXA-10 [14]. CTX-M enzymes are now predominant. They arose via multiple escape events of chromosomal β-lactamase-encoding genes (*bla*_klu_) from *Kluyvera* spp. [17–19], supported by the presence of transpositional units including IS*Ecp1* in CTX-M groups 1, 2, 9 and 25 or IS*CR1* in groups 2 and 9 [20]. Following initial reports in Europe [21], South America [22] and Japan [23], CTX-M enzymes have disseminated globally [24]. The group 1 enzyme CTX-M-15 is the most frequently identified, and dominates in many countries in Europe [25–30], Asia [31, 32], Africa [33–35] and the USA [36, 37]. Additional CTX-M mechanisms predominate in other locations. For example, the group 9 enzyme CTX-M-14 is the leading mechanism in *Escherichia coli* in some areas of Korea [38] and South America [39]. Until recently, CTX-M-14 was the major mechanism across China [40–42], but a steady increase in CTX-M-15 has also occurred [43–45].

**Table 1.** Classification of β-lactamases Adapted from Bush and Jacoby, 2010 [16].

Ambler molecular class	Bush–Jacoby group	Preferred substrate	Inhibited	Representative enzyme
A (serine penicillinases)	2a	Penicillins	+	PC1 from *S. aureus*
	2b	Penicillins, narrow-spectrum cephalosporins	+	TEM-1, TEM-2, SHV-1
	2be	Penicillins, narrow- spectrum and extended-spectrum cephalosporins	+	SHV-2 to SHV-6, TEM-3 to TEM-26, CTX-Ms, BEL-1, VEB-1, PER-1
	2br	Penicillins	−	TEM-30, SHV-72, SHV-19
	2c	Penicillins, carbenicillin	+	PSE-1
	2e	Extended-spectrum cephalosporins	+	FEC-1, CepA
	2f	Penicillins, cephalosporins, carbapenems	+/−	KPC-2, SME-1, NMC-A
B (MBLs)	3	Most β-lactams including carbapenems	−	IMP-1, VIM-1. NDM-1, CcrA and BcII, CphA, L1
C (cephalosporinases)	1	Cephalosporins	−	AmpC, CMY-2, ACT-1
D (oxacillinases)	2	Penicillins, cloxacillin	+/−	OXA-1, OXA-10
	2de	Extended-spectrum cephalosporins	+/−	OXA-11, OXA-15
	2df	Carbapenems	+/−	OXA-23, OXA-48

Genomic epidemiology demonstrates a number of widespread lineages including sequence type (ST)131, ST38, ST405 and ST10 in *E. coli* [46–49], and ST11, ST14 and ST15 in *Klebsiella pneumoniae* [32, 50, 51]. ST131, an extra-intestinal pathogenic *E. coli*, has undergone massive clonal expansion and is strongly associated with the global dissemination of the *bla*_CTX-M-15_ gene [47, 52, 53].

WGS has resolved ST131 into three clades, based upon the presence of marker alleles for the type 1 fimbriae, *fimH*. Clade A is associated with *H*41, clade B with *H22* and *H*30 is associated with clade C [54–58]. A clade C sublineage is the main driving force in the widespread dissemination of CTX-M-15 and fluoroquinolone resistance (FQR) in ST131 [55, 56, 59]. Clade C is identifiable by FQR mutations in *gyrA* (*gyrA1AB*) and *parC* (*parC1aAB*) genes, whereas clades A and B are predominantly fluoroquinolone susceptible [55]. Further segregation of clade C into C1 and C2 occurs depending upon the presence of *bla*_CTX-M-15_ [56, 59]. Prior to the emergence of C1 and C2, acquisition of elements including the GI-*pheV* genomic island [54] and the *H*30 allele [60] helped to prime ST131 for global success. C1 and C2 divergence and the development of FQR mutations is estimated to have occurred in the late 1980s, consistent with the introduction of fluoroquinolones for clinical use [54]. CTX-M-14, CTX-M-27, CTX-M-19, CTX-M-24 and CTX-M-55 have been identified in clade C [59]; however, CTX-M-15 is almost entirely restricted to C2 [55, 56, 59]. Bayesian analysis based upon CTX-M variant distribution also suggests *bla*_CTX-M-15_ emerged in ST131 following the introduction of extended-spectrum cephalosporins into clinical practice [59].

Plasmid movement between different species and lineages represents a major source of AMR. *bla*_CTX-M-15_ in ST131 is invariably associated with plasmids of incompatibility group F (IncF) [25, 59, 61–63], although presence on IncN [64], IncX [65] and IncI [66] plasmids has also been reported. Specific IncF plasmids have been associated with C2 isolates. This includes those with dual replicons, which complicates plasmid typing and broadens the plasmid host range [67, 68], additional AMR genes, gene cassettes, toxin/antitoxin systems and stability mechanisms, all of which may have influenced plasmid and clade success [57, 59, 69]. Architecture of the ST131 accessory genome, including plasmids, further supports clade-specific adaptations that have likely contributed to the success of ST131 [70]. Multiple clusters of variable accessory genome content within clade C suggest that clonal expansions of stabilized accessory gene profiles occur frequently, allowing generalization of this highly structured clone [59, 70].

## CPE

**Fig. 1.**
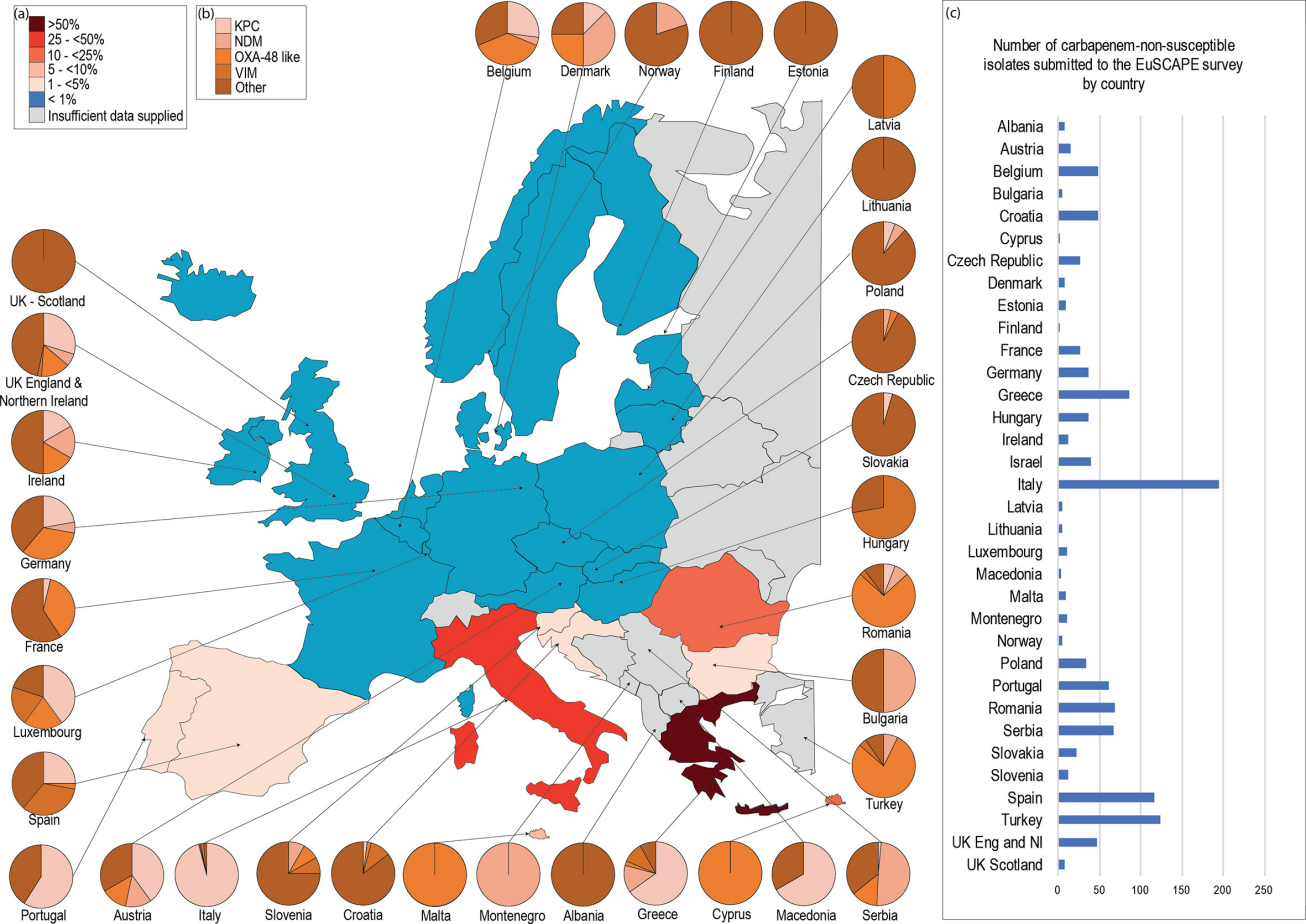
Composite figure demonstrating the prevalence and characteristics of carbapenem resistance in Europe. (a) Percentage of invasive isolates resistant to carbapenem antibiotics as determined by the European Centre for Disease Prevention and Control in the *Antimicrobial Resistance Surveillance in Europe 2015* report [11]. Each country is coloured according to the percentage of submitted *K. pneumoniae* isolates that were non-susceptible to doripenem, imipenem or meropenem. (b) Pie charts indicating the distribution of carbapenem-resistance mechanisms in *K. pneumoniae* isolates submitted to the EuSCAPE study [52]. Mechanisms are coloured according to the key. ‘Other’ mechanisms: no KPC, NDM, OXA-48 or VIM genes detected. (c) Overall number of *K. pneumoniae* isolates submitted by each participating country in the EuSCAPE study.

Rising ESBL-E prevalence correlates with increased carbapenem consumption [71, 72]; and appears to have driven the emergence and spread of carbapenem resistance, especially in *Enterobacteriaceae* [73]. Carbapenem resistance may be caused by different mechanisms, including inducible overexpression of chromosomal cephalosporinases, such as AmpC, combined with porin loss [74]. More problematic, however, is acquisition of carbapenemase genes via mobile genetic elements. The most frequently identified mechanism is the Ambler class A *K. pneumoniae* carbapenemase (KPC), followed by class B metallo-β-lactamases (MBLs) such as New Delhi MBL (NDM), and the class D OXA-type genes [75] (Table 2, Fig. 1).

**Table 2.** Carbapenem-resistance genes identified in *Enterobacteriaceae* *bla*_SME-1_, *Serratia marcescens* enzyme; *bla*_IMI_, imipenem-hydrolysing-β-lactamase; *bla*_KPC_, *K. pneumoniae* carbapenemase; *bla*_IMP_, active on imipenem; *bla*_NDM_, New Delhi MBL; *bla*_VIM_, Verona integron-encoded; *bla*_GIM_, German imipenemase; *bla*_KHM_, Kyorin Health Science MBL.

Gene	Species of origin*	Geographical origin† (year)	Active site	Ambler class	Location	Plasmid	No. of variants	Case
*bla*_SME-1_	*Serratia marcescens*	London, UK (1982)	Serine	A	Chromosomally encoded, SmarGI1 novel genomic island [250]		5	Mataseje *et al.* [250] – characterization of a novel genomic island
*bla*_IMI_	*Enterobacter cloacae*	California, USA (1984)	Serine	A	Chromosomally encoded in *Enterobacter cloacae*, IncF plasmid in *Klebsiella variicola* [251] and *Escherichia coli* [252]	IncF types [251, 252]	12	Rasmussen *et al.* [253] – characterization of first clinical IMI isolate
*bla*_KPC_	*Klebsiella pneumoniae*	North Carolina, USA (1996)	Serine	A	Tn*4401* [107]	Multiple [107, 254]	24	Munoz-price *et al.* [105] – description of an on-going UK outbreak
*bla*_OXA-48_	*Klebsiella pneumoniae*	Istanbul, Turkey (2001)	Serine	D	Tn*1999* [185]	IncL/M [185]	OXA-181, OXA-204, OXA-232, OXA-163	Potron *et al.* [175] – description of a clonal multi-country outbreak
*bla*_IMP_	*Serratia marcescens*	Aichi Prefecture, Japan (1991)	Zinc	B	Variable – chromosomal, class I integron [255]	IncA/C, IncH, IncL/M [255]	>52	Peleg *et al.* [256] – multi-genera dissemination of *bla*_IMP_ in Australia
*bla*_NDM_	*Klebsiella pneumoniae*	New Delhi, India (2008)	Zinc	B	Tn*125* [151]	Multiple [126, 151, 153]	16	Walsh *et al.* [145] – environmental spread of *bla*_NDM_
*bla*_VIM_	*Pseudomonas aeruginosa*	Verona, Italy (1997)	Zinc	B	Class I integrons, In*2*-Tn*402* [257]	IncHI2, IncI1 [257], IncN [258]	>46	Luzzaro *et al.* [259] – *bla*_VIM_ in multiple genera from one patient
*bla*_GIM_	*Pseudomonas aeruginosa*	North Rhine-Westfalia, Germany (2004)	Zinc	B	Not determined	Not determined		Rieber *et al.* [260] – emergence of *bla*_GIM_ in clinical samples
*bla*_KHM_	*Citrobacter freundii*	Tokyo, Japan (1997)	Zinc	B	Not determined	Not determined		Sekiguchi *et al.* [261] – first identification of *bla*_KHM_

*First species known to be reported in.

†First location reported in the literature.

Since its identification in the USA in 1996 [76], KPC has disseminated globally, has been reported to be present in more than 50 % of CPE in many countries, and in some cases 100 % of carbapenem-resistant *K. pneumoniae* [77–84]. The majority of KPC-encoding genes are seen in *K. pneumoniae* clonal group (CG)258, which includes the successful lineages ST258 and ST11 [85–87]. An example of this rapid dissemination can be seen in Greece. Following the first KPC isolation in 2007 [88], KPC had spread to most acute-care facilities within 2 years [89–92]. Most infections remain hospital-related, and associated with high mortality rates [6, 93–95]. Many early cases were epidemiologically linked to travel to high prevalence locations [96–101]; however, complex local transmission networks now signify endemicity [102, 103]. More than 20 KPC variants have been recognized, with *bla*_KPC2_ and *bla*_KPC3_ being the most abundant [79, 83, 85, 104–106]. The gene is located in isoforms of the 10 kb Tn*4401* transposon [107], of the Tn*3* transposon family [108, 109], and is associated with diverse plasmids including IncFIIK [87], IncI [110], IncN [111], IncL/M [112] and IncX [113].

Carbapenem-resistant lineages exhibit less diversity when compared to carbapenem-susceptible *Enterobacteriaceae* [114, 115] and lineages such as ST258 [112, 116, 117] and ST11 [84, 106] demonstrate clonal spread. However, in contrast to the clonality of ESBL lineages and predominance of a small number of globally disseminated epidemic lineages, carbapenemase genes and plasmids show increased transferability within and between species, lineages, STs and patients. This genetic mobility complicates the investigation of outbreaks [114, 118–120]. This has been observed more frequently in *E. coli* than other *Enterobacteriaceae*. The spread of carbapenem resistance displays increased diversity across STs, such as the large ST10 complex, rather than strong association with existing global epidemic lineages like ST131 [114, 121–123].

Non-clonal dissemination is also highly apparent in MBLs, especially NDM. These class B enzymes, which include NDM, GES, VIM and IMP, have also disseminated globally [124]. MBLs hydrolyse all β-lactams, are not inhibited by β-lactamase inhibitors, and their host bacteria often carry additional resistance mechanisms such as ESBLs [125–128]. First identified in a Swedish patient repatriated from a New Delhi hospital [129], most early cases had epidemiological links to the Indian subcontinent [130–143]. Epidemic spread and environmental contamination is evident in India, Pakistan and Bangladesh [144, 145], whilst sporadic cases or regional spread now occur on all continents [75, 84, 146, 147]. Clonal spread may occur during outbreaks [148, 149], but the high resolution of WGS enables tracking of varying *bla*_NDM_-positive plasmids including IncA/C, IncF, IncH, IncL/M, IncN and IncX types [113, 150–153], and fluctuating genomic contexts flanking the *bla*_NDM_ gene among non-clonal isolates [128, 151, 154–156]. The *bla*_NDM_ gene is chimeric following fusion with the aminoglycoside gene *aphA6* and lies downstream of either entire, truncated or remnants of the IS*Aba125* element [157].

*bla*_VIM_ genes were originally described in Italian *Pseudomonas aeruginosa* in the mid-1990s [158] and *Enterobacteriaceae* carrying *bla*_VIM_ are predominantly reported in Europe as occurring sporadically or in single hospital outbreaks [147]. Sporadic cases are also seen in Africa, Taiwan, Mexico, Saudi Arabia and the USA [159]. Since 2015, Hungary, Italy and Spain have reported inter-regional spread; however, as with other CPE mechanisms, *bla*_VIM_ is endemic in Greece [147]. More than 48 variants have been identified with *bla*_VIM-1_ and *bla*_VIM-2_ showing global dissemination [159]. *bla*_VIM_ genes are carried on variable class 1 integrons within multiple plasmid Inc types [159–161].

*bla*_IMP_ was the first described case of a transmissible carbapenemase gene [162]; however, large-scale epidemiological studies are lacking. The majority of *bla*_IMP_ isolates originate in the South Pacific [163] and Asia [164]. *bla*_IMP_ is found predominantly in *K. pneumoniae, E. coli* and *Enterobacter* spp. on class 1 integrons [165]. Integrons and their gene cassette combinations are variable and may show geographical correlations [164]. Despite being named due to imipenem resistance, certain variants of *bla*_IMP_, particularly *bla*_IMP-6_, actually exhibit low levels of imipenem resistance, which may lead to misidentification, and contribute to the lower detection rates of this mechanism [166, 167]. Genomic evidence is now emerging of this mechanism moving into epidemic *Enterobacteriaceae* such as *E. coli* ST131 [168, 169].

Finally, OXA-48 carbapenemases, first identified in 2001 in Turkey, are also a public-health threat [170–172]. Owing to their variable levels of carbapenem resistance, the spread of *bla*_OXA-48_ has been initially underestimated [173–175]. In parallel to *bla*_NDM_ and its Indian origins, *bla*_OXA-48_ was initially geographically linked to Turkey [171, 176]. However, since 2015, multiple countries have inter-regional spread and *bla*_OXA-48_ is endemic in Malta and Turkey [147]. Further afield, extensively drug resistant (XDR) strains co-harbouring *bla*_NDM_ and *bla*_OXA-48_ have been identified in the Middle East [177, 178], and *bla*_OXA-48_ strains have emerged in Canada [173], Algeria [179] and Korea [180]. *Shewanella* spp. may be the natural progenitors of *bla*_OXA-48_ genes [181], which now predominantly appear in *K. pneumoniae, E. coli* and *Enterobacter* spp. [173, 182, 183]. *bla*_OXA-48_ is associated with the Tn*1999* transposon, which is composed of two copies of IS*1999* bracketing the gene [184, 185]. The majority of *bla*_OXA-48_ genes are associated with *Tn*1999 or the variants Tn*1999.2* [171], Tn*1999.3* [186] and Tn*1999.4* [187]. Tn*1999.4* is a mosaic of Tn*1999* and a second transposon, Tn*2015*, which additionally carries *bla*_CTX-M-15_ [187]. In contrast to other CPE genes, dissemination of *bla*_OXA-48_ is associated with a single, successful IncL/M plasmid into which the Tn*1999* transposon has inserted [173, 174, 178, 185, 187–193].

A variant of *bla*_OXA-48_, *bla*_OXA-181_, has also begun to disseminate among *Enterobacteriaceae* and appears to be establishing in the Indian subcontinent, South Africa and Singapore, or in patients epidemiologically linked to these areas [194–199]. Recently, the first cases of likely patient-to-patient transmission have also been reported [200, 201]. *bla*_OXA-181_ has been identified on a non-self-conjugative ColE2 plasmid in association with IS*Ecp1* and the Tn*2013* transposon [198]. Additionally, *bla*_OXA-181_ has been identified in the same strains as *bla*_NDM_ genes, reflecting its prevalence in India [201, 202], and now in a conjugative plasmid [202], suggesting widespread dissemination may occur in the future.

## The continued threat of AMR

The impact of antibiotic consumption is reflected in geographical variations of CPE and ESBL-E prevalence. Countries with high antibiotic consumption rates, such as Turkey, Tunisia, Algeria, Greece and Romania [71], have particularly high rates of multidrug-resistant (MDR) bacteria [11, 147]. Overuse of particular antibiotic classes also affects MDR organisms, such as in Greece where high cephalosporin use [203] is paralleled by high levels of ESBL-E [11]. Travel to endemic regions also may be having a global impact following acquisition of MDR pathogens by travellers [204–208].

A particularly concerning issue, especially in Asia, is transferable colistin resistance [209]. Increased carbapenem resistance has resulted in an increase in the use of polymyxins (e.g. colistin) to treat XDR pathogens [71, 210]. We are now faced with the dissemination of genes conferring resistance to these drugs, which are frequently co-located with additional resistance genes, leaving some infections almost untreatable [211–214]. Following the first publication of the transferrable colistin-resistance gene, *mcr-1* [209], screening has demonstrated global existence of *mcr-1* in food, animal and human samples [215, 216]. Following the association of *mcr-1* with IS*Apl*-1 of the IS*30* family and formation of the composite transposon Tn*6330*, *mcr-1* and its genetic environment has stabilized [217–219]. It is now beginning to spread across multiple plasmid types [214, 220–224]. The ancestral mobilizable state of *mcr-1* is more frequently identified in agricultural isolates than human isolates, particularly those in China, supporting the theory of an animal origin [209, 225–227]. Colistin is ubiquitous in food-animal production [228], but its use as a growth promoter has been banned in the European Union since 2006 and in China since 2016 [229, 230]. This may begin to ease the antibiotic selection pressure; however, it is difficult to speculate how this may affect the human situation as stabilization and dissemination of the gene into conjugative plasmids has already occurred.

## Conclusion

Antimicrobial stewardship as a strategy to reduce AMR is high on policy agendas in many countries [231–235] and a positive impact on the prevalence of MDR pathogens is beginning to show [236, 237]. Continued strategy development is still required; accepted international definitions and guidelines are yet to be adopted, particularly those suitable for low-to-middle income countries [238]. With the inception of the ‘One Health’ initiative [233, 239, 240], consideration should also be given to antimicrobial prescription in primary care [30, 210, 241, 242], poorly regulated community antimicrobial use [243–246] and agricultural antimicrobial use [239, 247–249].

The ability of CPE and ESBL-E to evolve and adapt rapidly due to antibiotic selective pressures is one of the biggest threats to medical care. An international, multi-disciplinary approach is urgently required to tackle this global threat. Pressing issues include improving surveillance to recognize the importance of mobile AMR elements and increasing the drive to move rapid, high-resolution diagnostics, such as WGS, from the research environment into routine clinical practice. A proactive approach involving all users of antimicrobials is imperative to prevent a return to the pre-antibiotic era.
