# Corrigendum: Extended-spectrum β-lactamase-producing and carbapenemase-producing *Enterobacteriaceae*

**DOI:** 10.1099/mgen.0.000218

**Published:** 2018-09-28

**Authors:** Hayley Wilson, M. Estée Török

**Affiliations:** ^1^​ Department of Medicine, University of Cambridge, Addenbrooke's Hospital, Cambridge CB2 0QQ, UK; ^2^​ Cambridge University Hospitals NHS Foundation Trust, Cambridge, UK; ^3^​ Clinical Microbiology and Public Health Laboratory, Public Health England, Cambridge, UK

There was an error in the legend of Fig. 1 in the published article. A new reference [262] replaced reference [52] and was also added to the last sentence. The legend should read as the following:

Fig. 1. Composite figure demonstrating the prevalence and characteristics of carbapenem resistance in Europe. (a) Percentage of invasive isolates resistant to carbapenem antibiotics as determined by the ECDC in the *Antimicrobial resistance surveillance in Europe 2015 report* [11]. Each country is coloured according to the percentage of submitted *K. pneumoniae* isolates that were non-susceptible to doripenem, imipenem or meropenem. (b) Pie charts indicating the distribution of carbapenem resistance mechanisms in *K. pneumoniae* isolates submitted to the EuSCAPE study [262]. Mechanisms are coloured according to the legend in box B. ‘Other’ mechanisms: no KPC, NDM, OXA-48 or VIM gene detected. (c) Overall number of *K. pneumoniae* isolates submitted by each participating country in the EuSCAPE study [262].
